# A power law global error model for the identification of differentially expressed genes in microarray data

**DOI:** 10.1186/1471-2105-5-203

**Published:** 2004-12-17

**Authors:** Norman Pavelka, Mattia Pelizzola, Caterina Vizzardelli, Monica Capozzoli, Andrea Splendiani, Francesca Granucci, Paola Ricciardi-Castagnoli

**Affiliations:** 1 Department of Biotechnology and Bioscience, University of Milano-Bicocca, Piazza della Scienza 2, 20126 Milan, Italy

## Abstract

**Background:**

High-density oligonucleotide microarray technology enables the discovery of genes that are transcriptionally modulated in different biological samples due to physiology, disease or intervention. Methods for the identification of these so-called "differentially expressed genes" (DEG) would largely benefit from a deeper knowledge of the intrinsic measurement variability. Though it is clear that variance of repeated measures is highly dependent on the average expression level of a given gene, there is still a lack of consensus on how signal reproducibility is linked to signal intensity. The aim of this study was to empirically model the variance versus mean dependence in microarray data to improve the performance of existing methods for identifying DEG.

**Results:**

In the present work we used data generated by our lab as well as publicly available data sets to show that dispersion of repeated measures depends on location of the measures themselves following a power law. This enables us to construct a power law global error model (PLGEM) that is applicable to various Affymetrix GeneChip data sets. A new DEG identification method is therefore proposed, consisting of a statistic designed to make explicit use of model-derived measurement spread estimates and a resampling-based hypothesis testing algorithm.

**Conclusions:**

The new method provides a control of the false positive rate, a good sensitivity vs. specificity trade-off and consistent results with varying number of replicates and even using single samples.

## Background

DNA microarrays have become common tools for monitoring genome-wide expression in biological samples harvested under different physiological, pathological or pharmacological conditions. One of the most challenging problems in microarray data analysis is probably the identification of differentially expressed genes (DEG) when comparing distinct experimental conditions. In spite of its biological relevance, there is still no commonly accepted way to answer this question.

An ideal DEG identification method should limit both false positives, i.e. genes wrongly called significant (type 1 errors), and false negatives, i.e. genes wrongly called not significant (type 2 errors). To this end, understanding how gene expression values measured in replicated experiments are spread around the true expression level of each gene, would help to distinguish biologically relevant gene expression changes from fluctuations due to different sources of variability that are unrelated to the biological phenomenon under investigation. Measurement error estimates can be obtained in two ways: either by empirically inferring noise from highly replicated data or by deducing noise from a theoretical error model [1]. Especially when the experimental design requires the investigation of a high number of conditions, the former strategy is not always feasible, because of the high cost of these experiments or due to the availability of biological material. In addition, there is still a lack of consensus on how gene expression values from replicated experiments should be theoretically distributed, which restricts the application also of the latter strategy.

The most widely used methods for identifying DEG range from purely empirical filtering techniques (e.g. selecting genes that show a fold change higher than a fixed threshold) to more sophisticated statistical tests such as the signal-to-noise ratio described by Golub *et al*. [2] or the Significance Analysis of Microarrays (SAM) method by Tusher *et al*. [3]. While empirical filtering techniques rely on arbitrarily chosen thresholds and are unable to provide any type of control on the significance of the results, the more sophisticated statistical tests usually need a high degree of replication in the data to accurately measure gene-specific variability.

In the past years various authors have proposed competing error models for microarray data from which discordant implications for the variance versus mean dependence can be deduced. Chen *et al*. [4] first proposed a simple Gaussian model, more recently Ideker *et al*. [5] and Li and Wong [6] introduced two-component models containing a multiplicative and an additive error term. All of these models implicitly or explicitly assume a constant coefficient of variation (CV), implying that standard deviation should vary proportionally with the mean. More recently, Rocke and Durbin [7] proposed a variation of the two-component model from which they derived that variance of repeated microarray measures is a quadratic function of the mean. Dealing specifically with spotted cDNA microarray technology Baggerly *et al*. [1] proposed a beta-binomial model, from which it can be derived that variance is a second-order polynomial function of the mean. Unfortunately, most of these models are based on theoretical assumptions that have been verified on simulated data or on data sets consisting of small numbers of replicates. More recently, Tu *et al*. [8] empirically modeled the variance versus mean dependence from a data set consisting of ten replicated oligonucleotide microarray experiments. According to the authors, the variance of the genes should decay exponentially with the mean, but only for moderately expression values. Taken together, all these aspects could limit the applicability of these error models.

Independently from the choice of the error model, another point that remains to be faced is on how to estimate residual error. A discussed by Wright *et al*. [9] the possibilities range between two extremes: either obtaining a single variance estimate across all genes or obtaining a gene-specific residual variance. In the same paper a hybrid approach is proposed in which information from all genes is used to fit a single linear model from which the gene-specific variance estimates can be deduced. In the present work we chose to follow an approach similar to the latter.

The aim of this study was to use highly replicated microarray data to empirically determine the true variance versus mean dependence that exists in this type of data. This knowledge enabled the proposal of PLGEM as a simple but powerful error model. We fitted the proposed model on various data sets without pre-filtering the data, deriving an improved test statistics and identifying DEG even in data sets with very little number of replicates.

## Results

### Variance versus mean dependence

**Figure 1:**
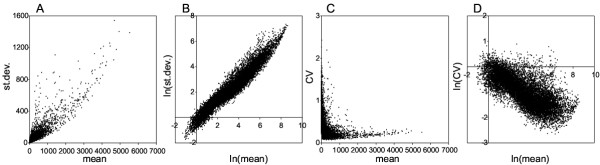
**Relationship between measurement variability and mean expression level**. For each of the 12488 probe sets displayed on Affymetrix MG-U74Av2 chips the standard deviation (st. dev.) or the coefficient of variation (CV = st. dev. / mean) is plotted against the mean in either linear or log-log plots based on absolute expression values derived from the 16iDC data set.

The relationship between measurement variability and average expression values was investigated by means of scatter plots where different measures of spread were displayed against different measures of location. For each gene absolute or relative standard deviation was plotted against the mean expression value in either linear or log-log plots using data from the 16iDC data set (Figure 1). Independently from the choice of standard deviation or inter-quartile range as the estimate of spread and of mean or median as the location estimate we obtained qualitatively similar plots (data not shown). Log-log plots of both absolute and relative spread estimates revealed a strikingly linear dependency, indicating that measurement spread could depend on signal location following a power law.

### The power law global error model

Based on the previous observation, we chose to empirically model measurement noise through linear regressions:



where *s *and  respectively represent standard deviation and mean of repeated measures. Error term *ε *is the realization of a random variable E that we will show later to be normally distributed as assumed when fitting a linear model. Inspired by the previous experimental observations we propose the following power law global error model (PLGEM):



Model parameters *α *and *β *can be estimated from linear regression coefficients in **1 **in a straightforward way:

*α *= *e*^*c *^    *eqn*. 3

*β *= *k *    *eqn*. 4

### PLGEM fitting method

Instead of performing a simple linear fit through the whole set of points, we preferred to implement a method that could provide improved model robustness by partitioning the data to gain local estimates of spread as in Mutch *et al*. [9]. Most importantly, this method should also provide the possibility to choose different levels of confidence when modeling the spread of the data. Note that Mutch *et al*. [9] proposed to model within-replicates fold changes as a function of average expression using a model that was very different from PLGEM. Therefore, the following algorithm was applied:

• Rank genes according to their ln() value and subdivide the overall expression range into a given number p of partitions containing an equal number of ranked genes.

• Choose a "modeling quantile" q and determine for all the genes contained in each partition a single "modeling point" with median of ln() values as the x-coordinate and q-th quantile of ln(*s*) values as the y-coordinate.

• Finally, find a linear fit through the set of p modeling points using the least-squares method and obtain a slope k and an intercept c of the resulting regression function.

Thus, for all possible combinations of p and q a slope k_p,q_, an intercept c_p,q _and a correlation coefficient r^2^_p,q _can be obtained. Performance of this modeling method was tested also using different combinations of partitions, in the range between 5 and 500, and quantiles, ranging from 0.01 to 0.99 (Supplementary Table 1). For all 77 analyzed combinations of p and q regression lines gave good fit with the modeling points, with an adjusted r^2 ^that was always very close to 0.99. In addition, all regression lines were strikingly parallel as judged by their slopes: 0.81 ± 0.068 (mean ± sd). The reason for not considering p > 500 was that above this number we empirically revealed a poorer modeling quality in terms of correlation coefficients (data not shown), most likely due to the decrease of the number of data points contained in each partition.

**Table 1:** Effect of the presence of DEG when applying the classic permutation strategy to the PLGEM-STN statistic.

	FPR vs. significance level						estimated vs. observed FDR					
	
	including DEG			excluding DEG			including DEG			excluding DEG		
	
# of genes in data set	slope	intercept	adj. R^2	slope	intercept	adj. R^2	slope	intercept	adj. R^2	slope	intercept	adj. R^2
22300	1.690	3.070	0.856	0.871	-0.114	0.938	0.187	-0.383	0.314	1.090	-0.692	0.826
10000	1.710	2.470	0.881	0.888	-0.083	0.931	0.224	-0.247	0.433	1.040	-0.555	0.824
5000	1.460	1.270	0.880	0.877	-0.147	0.934	0.093	-0.228	0.067	1.080	-0.425	0.836
2500	1.590	1.180	0.888	0.864	-0.155	0.939	0.092	-0.176	0.135	1.110	-0.348	0.853
1500	1.670	0.935	0.908	0.876	-0.166	0.948	0.078	-0.122	0.170	1.130	-0.182	0.880
1000	1.720	0.689	0.874	0.944	0.002	0.956	0.038	-0.122	0.082	0.991	-0.226	0.897
500	1.900	0.263	0.864	0.857	-0.255	0.956	0.062	-0.030	0.307	1.160	0.038	0.909
200	2.490	-0.059	0.875	0.905	-0.378	0.946	0.064	-0.012	0.426	1.050	0.233	0.914

Eight data sets with different percentages of DEG were constructed from the Latin Square data set by keeping the 62 known spiked-in probe sets, but randomly removing increasing amounts of the remaining probe sets reaching the total indicated in the first column. The null distributions of the PLGEM-STN statistics were evaluated through the classic permutation strategy either including or excluding DEG. A wide range of significance levels was used to select DEG and correlation between the FPR and the significance level or between estimated and observed FDR was evaluated through linear regressions in log-log plots. Table reports slopes, intercepts and adjusted r^2 ^of linear models. See text for details on estimation of FDR.

As a straightforward application of this modeling method, PLGEM could be fitted at the 50^th^-percentile to obtain a central tendency of standard deviation to be used for improving test statistics (see next section). Another application of this method could be to fit PLGEM at the 5^th^- and at the 95^th^-percentile of standard deviation, to consequently find the limits of the corresponding 90% empirical confidence interval of standard deviation.

**Figure 2:**
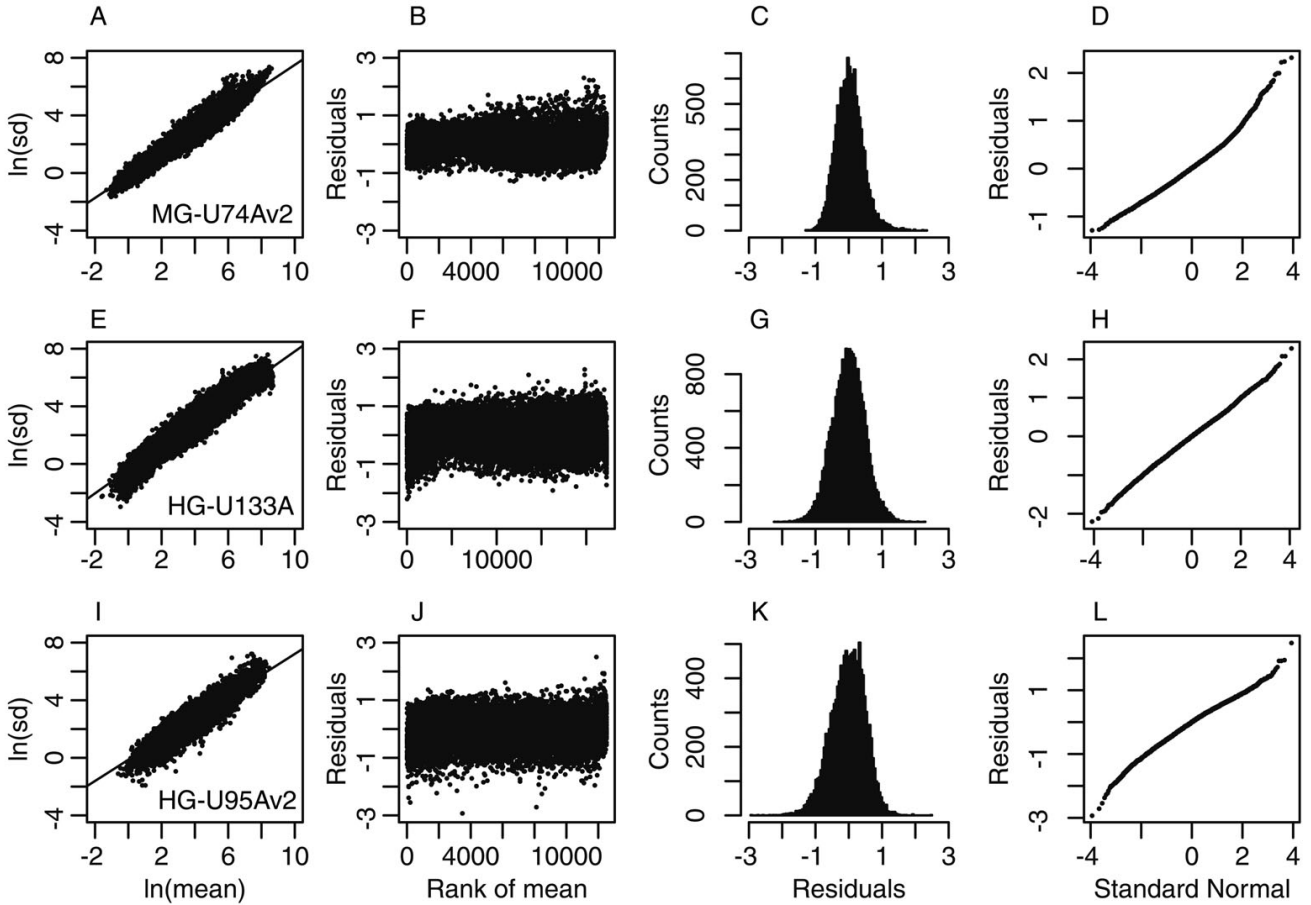
**Analysis of residuals of PLGEM fitted on three different real-life data sets**. (A) PLGEM is fitted on the 16iDC data set (MG-U74Av2) following the method described in the text, setting p = 10 and q = 0.5. (B) Model residuals are plotted as a function of the rank of the mean absolute expression level. (C) Distribution of pooled residuals. (D) The quantiles of the distribution of pooled residuals are plotted against the quantiles of a standard normal distribution. The same procedure is applied to the Leigh syndrome data set (HG-U133A, panels E-H) and to the Muscle biopsies data set (HG-U95Av2, panels I-L).

In order to verify the feasibility of the former application, fitting of PLGEM on real-life data as well as distribution properties of the random variable E were investigated by analyzing the residuals of the model, i.e. differences between observed and expected values:



Figure 2A–D shows distribution of residuals *ε*_g _computed from the 16iDC data set and its dependency on the rank of mean expression values. Figure 2E–L summarizes model validation on other two completely unrelated high-density oligonucleotide microarray data sets, the HG-U133A Leigh syndrome data set (Figure 2E–H) and the HG-U95Av2 Muscle biopsies data set (Figure 2I–L). For each tested data set an individual model was fitted and a distinct set of parameters *α *and *β *was determined. In all of the three independent data sets measurement variability could be accurately modeled through equation **2**, with a power coefficient *β *that was always between 0 and 1 and a random variable E that appeared to be normally distributed with zero-mean and constant standard deviation over the whole range of expression values. Of course, these findings were eventually expected only for q = 0.5, and their occurrence demonstrated a goodness of fit of PLGEM on a series of unrelated real-life data sets.

### Improved test-statistics for detecting differential expression

In order to identify DEG, we implemented the following general algorithm derived from the framework of statistical hypothesis testing, in which we test against the null hypothesis of non-differential expression. First of all, we chose to implement as the test statistic the signal-to-noise ratio (STN) already used by Golub *et al*. [2], because it explicitly takes unequal variances into account and because it penalizes genes that have higher variance in each class more than those genes that have a high variance in one class and a low variance in another [11]:



where in the original version  and  represent, respectively, the mean of the replicated expression measures of gene g in condition 1 and 2, whereas  and  are the corresponding standard deviations. Instead, we propose to use model-derived standard deviation estimates predicted by PLGEM in equation **2 **for the corresponding signal mean, rather than data-derived standard deviation values calculated independently from the few data points that are usually available for every single gene.

**Figure 3:**
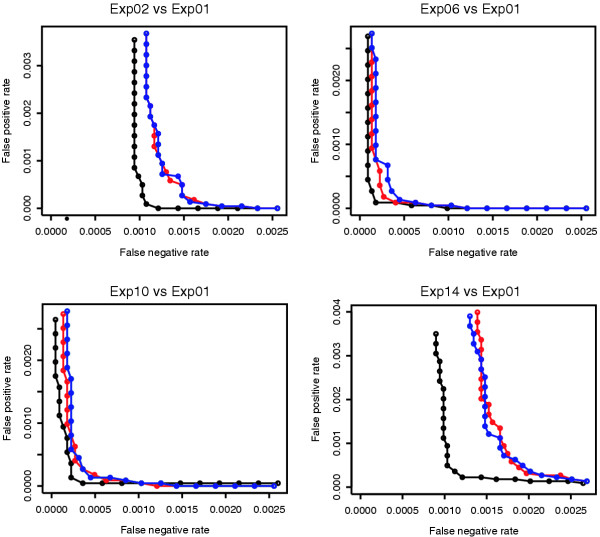
**Performance of PLGEM-STN in ranking differentially expressed genes**. ROC plots were used to compare the sensitivity vs. specificity trade-off of the following three statistics: PLGEM-STN (black), CLASSIC-STN (blue) and SAM-STAT (red). SAM-STAT values were obtained using the R package "siggenes" [21]. Absolute values of the corresponding statistics were sorted in decreasing order, first **n **genes were selected (where **n **ranged from 5 to 200) and false positive and false negative rates were evaluated on the HG-U133A Latin Square dataset. Note that, while the transcripts in Exp02 (Exp14) are spiked-in at twice (half) the concentration than in Exp01, in both Exp06 vs. Exp01 and in Exp10 vs. Exp01 comparisons the nominal fold-change of spiked-in transcripts ranged from 32 to 512.

The improvement of the test statistic in ranking DEG was evaluated as done by Broberg [12] through receiver operator characteristic (ROC) plots on the HG-U133A Latin Square data set, where there is an *a priori *knowledge on the truly differentially expressed transcripts. ROC plots investigate the relationship between false positive rates (FPR) and false negative rates (FNR) at different significance levels; in this way the performance of the PLGEM-derived STN statistic (PLGEM-STN) has been compared with the original STN statistic (CLASSIC-STN) and the statistic implemented in the commonly accepted Significance Analysis of Microarrays (SAM) DEG identification method (SAM-STAT). To this purpose Exp01 of the Latin Square was taken as the baseline to which the remaining 13 experiments were compared. For each comparison absolute values of each statistic were ranked in decreasing order and first **n **genes selected (where **n **ranged from 5 to 200). Figure 3 summarizes results only for the most informative comparisons, but in each tested comparison analysis PLGEM-STN was at least as good as the other two statistics for each tested value of **n **(data not shown). In addition, the ROC curve of PLGEM-STN always had the shortest distance from origin, indicating that it resulted in the best trade-off between sensitivity and specificity. Interestingly, improved sensitivity was observed especially when the nominal fold change was particularly low (see Exp02 vs. Exp01 and Exp14 vs. Exp01).

**Figure 4:**
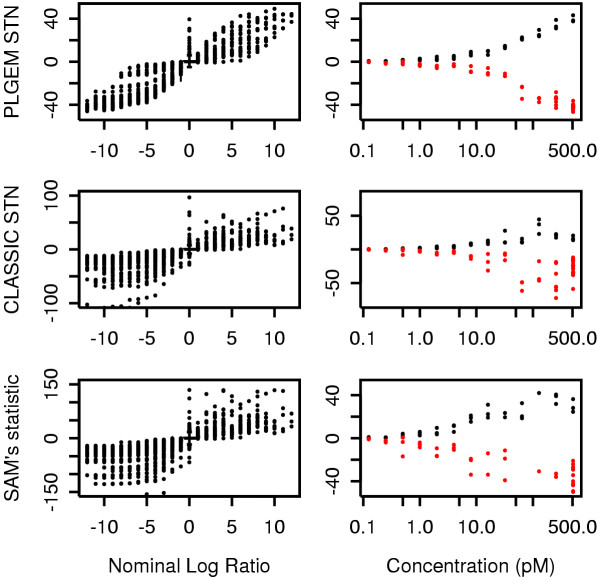
**Correlation between the value of PLGEM-STN and the nominal concentration variation in comparison to competing statistics**. Exp01 of the Latin Square data set was taken as the baseline to which the remaining 13 experiments were compared. The observed values of the indicated statistics are plotted against the nominal log ratio, deduced from the known spiked-in concentrations (left panels). A nominal log ratio of 0 is assumed for the remaining transcripts and a box-plot of their corresponding values of the indicated statistics is superimposed to the plot. For those cases where one of the two known spiked-in concentrations is 0, the value of the statistic is instead plotted against the non-null concentration (right panels). Red and black dots represent transcripts that are present in Exp01 or in the remaining 13 experiments, respectively.

Apart from discriminating between significant and not significant gene expression changes, an optimal test-statistic should additionally provide an accurate quantification of the actual degree of differential expression. Figure 4 shows that PLGEM-STN outperforms the competing statistics in correlating the value of the statistic with the nominal concentration variation of the known Latin Square DEG; this was particularly true for the most extreme variations.

### Identification of differentially expressed genes

#### A resampling-based method for estimating the null distribution

Though ranking of genes based on the absolute value of their test-statistic has been proven to be an effective method for selecting DEG, an even more useful way would be to compare the observed statistic with its null distribution (the distribution of values of the statistic that are expected by chance for a not differentially expressed gene), in order to control the FPR.

A classic approach to empirically obtain the null distribution of a test-statistic is running a series of random permutations of the chip indexes of the full data set and re-computing the test-statistics at each permutation. Permutated test-statistics can then be pooled and significance thresholds (i.e. expected false positive rates) are found as specific quantiles of the null distribution.

Nevertheless, we can foresee that the classic permutation strategy may not be optimal for estimating the actual FPR when the test-statistic makes use of a global error model such as PLGEM. We can in fact hypothesize that measurement spread of DEG may not be accurately described by means of a global error model that was designed to describe signal variability in absence of differential expression. To test this hypothesis we compared the correlation between the expected significance level and the observed FPR using PLGEM-STN and the classic permutation strategy either including or excluding DEG during the permutation step. To this end, data sets containing different percentages of DEG were obtained by merging the 62 known DEG of the Latin Square data set with differently sized random samples of not DEG extracted from the same data set. As predicted, the presence of DEG during the permutation step caused the significance level to be less correlated with the observed FPR and this correlation worsened with increasing percentages of DEG (Table 1). This lack of correlation was dramatically amplified when expected and observed numbers of false positives were divided by the number of selected genes to obtain an oversimplified estimate of the false discovery rate (FDR) and the observed FDR. Conversely, when DEG were omitted during the permutation step the correlation between estimated and observed FPR or estimated and observed FDR was sensibly higher for each tested percentage of DEG. We hereby by no means claim that this FDR estimate is the most accurate. A more appropriate relationship between FPR and FDR can be found in the paper by Storey and Tibshirani [13]. Nevertheless, the explicit control of the FDR goes beyond the scope of the present paper.

Since in real-life data sets true DEG are unknown in advance, we propose the following resampling-based method to obtain the null distribution of not DEG when comparing n_1 _replicates of condition A with n_2 _replicates of condition B:

• Artificial condition A* is obtained by randomly sampling with replacement n_1 _indexes corresponding to the replicates of only one experimental condition. If available, chose the condition with the highest number of replicates;

• Similarly sample n_2 _values from the same set to obtain indexes of artificial condition B*;

• Compute resampled test-statistics between A* and B* at each cycle.

**Figure 5:**
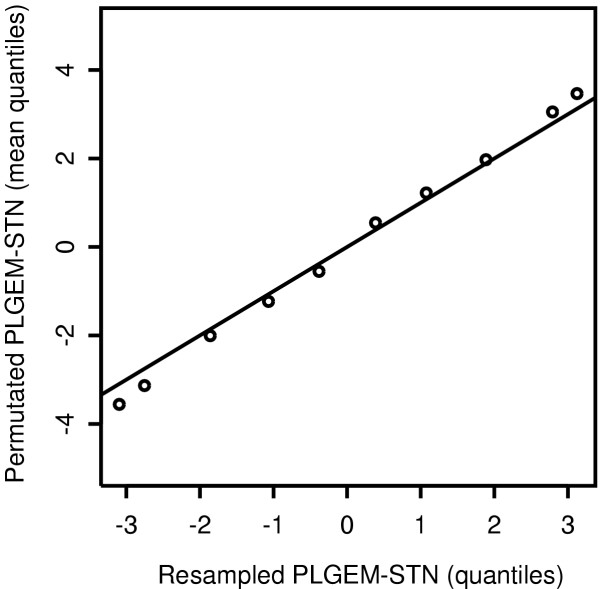
**Comparison of two methods for inferring the null distribution of the PLGEM-STN statistic**. The classic permutation strategy (excluding DEG) was performed for each comparison in the Latin Square data set and the quantiles of the distribution of PLGEM-STN values were averaged over the 13 comparisons. The mean quantiles of the permutated statistics are plotted against the quantiles of the distribution of PLGEM-STN values obtained through the proposed resampling approach performed on the same data set but including DEG.

The previous resampling should be repeated a sufficiently large number of times – as large as possible compared to the total number of possible combinations and compatibly with available computational resources – and the resampled test-statistics finally pooled. In our opinion resampling the expression values from only one experimental condition, rather than permutating indexes of both conditions, makes more sense with this particular statistic, because in this way we avoid merging true and false null hypothesis. Note that when more than one condition (all with the same number of replicates) are to be compared to a common baseline, the distribution of resampled test-statistics needs to be determined only once, obviously providing a computational advantage. As a test of substantial equivalence between this resampling method and the classic permutation strategy (excluding DEG), we compared the distribution of the permutated and of the resampled PLGEM-STN test-statistics in Q-Q plots. The distribution of the PLGEM-STN resampled from Exp01 of the Latin Square data set was almost identical with the distributions of permutated PLGEM-STN obtained with the classic strategy from each comparison with the remaining 13 experimental conditions (data not shown). Figure 5 shows that the quantiles of the resampled PLGEM-STN values have a good concordance with the mean quantiles of the classically permutated statistics averaged over the 13 comparisons, implying that no differences are expected also in the gene selection step.

In accordance with the previous observations, the ROC curve of the resampling method applied to the PLGEM-STN statistic was not significantly different from the ROC curve of the classic permutation strategy (excluding DEG) applied to the same statistic on the Latin Square data set (data not shown). Conversely, ROC curves of the classic permutation strategy (including DEG) applied to the CLASSIC-STN statistic and of the SAM method gave poorer performance similarly to the results in Figure 3 (data not shown).

#### Increased robustness to varying number of replicates

**Figure 6:**
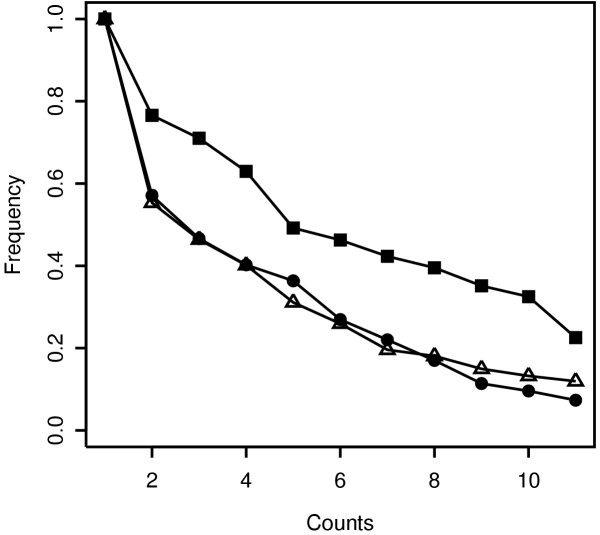
**Consistency of findings when different replicates or numbers thereof are analyzed**. Ten reduced data sets were constructed by removing all possible combinations of 1 or 2 replicates of LPS-stimulated DC from the data set. The plot shows the cumulative frequency at which the probe sets are consistently selected in the 16iDC+LPS and in the ten reduced data sets by the following methods: the resampling approach applied to PLGEM-STN (filled squares), the permutation strategy applied to CLASSIC-STN (filled circles) and the SAM method (open triangles). In case of PLGEM, a single model was fitted on the common 16iDC baseline data set. The cumulative frequencies are normalized with respect to the total number of probe sets identified by the corresponding method.

Another appealing feature of an optimal DEG identification method is that it should provide consistent results when different replicates of a same data set or different numbers thereof are analyzed. We therefore compared the performance of our resampling approach applied to the PLGEM-STN statistic (method 1) with SAM (method 2) and with the classic permutation strategy applied to the CLASSIC-STN statistic (method 3). The number of available replicates for each experimental condition in the Latin Square data set was unfortunately too small to investigate this particular task. We therefore took advantage of the 16iDC+LPS data set, where the first sixteen columns can be considered as the baseline condition for the remaining four experimental replicates. We then constructed a series of reduced data sets in which the baseline columns were kept constant while all possible combinations of 1, 2 or 3 replicates of LPS-stimulated DC were systematically deleted from the 16iDC+LPS data set, reaching a total of fifteen distinct data sets including the original one. Since methods 2 and 3 are not applicable on the four reduced data sets containing single samples for the LPS experimental condition, only the eleven data sets with at least two replicates were used for comparison purposes. Since the sixteen baseline columns are identical in each reduced data set, PLGEM parameters were determined only once on this common baseline condition. Significance levels used by each method in all eleven data sets were empirically selected in order to achieve a similar number of significant genes (ca. 500 probe sets) in the full data set, i.e. the one containing all available replicates. Thus, for each method eleven lists of identified DEG were obtained and the consistency between these lists was evaluated by counting the number of times each probe set was selected, giving a probes set count between 1 and 11. In Figure 6 we compared the three distinct cumulative frequency curves for each method, which show the percentage of identified DEG that were selected at least a given number times. While method 2 and 3 gave similar results, the method proposed in the present work identified a larger number of probe sets in a larger number of lists.

We finally evaluated the possibility of applying our method also to data sets where one of the experimental conditions was investigated only with a single sample without replication. To this end, we used the remaining four reduced data sets that could not be used in the previous comparison. In this case, the same PLGEM parameters derived from the sixteen baseline columns were applied to each of the single LPS-treated DC sample to obtain an estimate of standard deviation associated to each gene expression value, treated here as if it was a mean value from a larger group of values. Interestingly, when results obtained through this procedure were compared to the previously described results a comparable number of DEG was identified and only one probe set was newly detected in comparison to the previously identified ones (data not shown), arguing for a good consistency of results.

## Discussion

### PLGEM accurately describes GeneChip data variability

In the present work we described a new global error model for microarray gene expression data that describes measurement variability with the same degree of accuracy over the whole dynamic range of values and that can be fitted at any desired quantile of spread. PLGEM has proven to correctly model signal standard deviation, in spite of the presence of different sources of variability, e.g. biological variability as well as the use of different target preparation protocols or of different chips. Moreover, PLGEM has shown to be able to deal with the great variability that exists at low expression levels while at the same time considering the significant relative reproducibility of highly expressed genes. Previously proposed error models assumed that measurement spread depended on signal location following different mathematical relationships, but none of them was based on a power law thus far. Analysis of the residuals showed a good fit of PLGEM to a number of high-density oligonucleotide microarray data sets, with model parameters being very similar to each other even when dealing with RNA samples coming from completely different biological sources and analyzed on different array layouts. This suggests that PLGEM could represent a general Affymetrix GeneChip measurement noise model. Even though scaled MAS5 Signals gave satisfactory modeling results, a further improvement could be achieved by using other emerging gene expression indices [6,14] or more sophisticated normalization techniques, e.g. quantile normalization [15]. Interestingly, if the same evaluation of sensitivity vs. specificity using ROC plots on the Latin Square data set was done using GCRMA expression values [16], the results were even more striking than using MAS5 Signals (data not shown). Further studies will be needed to assess if PLGEM is also able to deal with data coming from microarray technologies others than Affymetrix GeneChips.

Interestingly, model parameter *β *was found to be quite stable and comprised between 0 and 1 in all analyzed data sets. It is noteworthy that for *β *∈ (0:1) absolute variability increases with growing expression values, while relative variability decreases (compare panel B with panel D of Figure 1). On the other hand, none of the models mentioned in the background section seem to agree with these experimental observations. Formal statistical reasoning could unravel the underlying theoretical error model that leads to the power law relationship that was observed to be at the basis of the variance versus mean dependence in replicated microarray data.

### A PLGEM-based method successfully detects differential expression

In spite of the lack of a theoretical statistical model, the empirical model presented here has proven its applicability in the identification of DEG, providing improved results under a wide range of different testing conditions. In comparison to other commonly used DEG identification methods, the proposed approach demonstrated improved specificity and sensitivity on the Latin Square data set and robustness to decreasing number of replicates on the 16iDC+LPS data set. The good performance of our proposed method is reasonably due to the fact that it relies on a global error model. As an example, when the classic permutation strategy is applied to the CLASSIC-STN statistic or when the SAM method is used, the selected genes are apparently more dependent on the number and identity of the replicates than when our proposed approach is used. We hypothesize that, when no error model is assumed and a small number of replicates is present in the data set, the probability of observing for some genes coincidently very similar (or very dissimilar) values increases, thus leading to an underestimation (or overestimation) of the standard deviation and a consequent overestimation (or underestimation) of the test statistic, finally leading to false positives (or false negatives).

Interestingly, when the performance of our method was compared on a data set of DC stimulated for 24 hours with LPS, SAM showed a decreased sensitivity in identifying down-regulated genes when the number of LPS replicates was low (data not shown). Under these experimental conditions DC undergo a process known as maturation, which is a specialized form of cellular differentiation, for which both up- and down-regulation of gene expression is expected [17,18]. We speculate that SAM did not select these genes, because of the combination of two effects. First of all, down-regulated genes are expected to have lower and therefore intrinsically more variable expression values in the four LPS replicates than in the sixteen replicates of immature DC. When, in addition, the number of LPS replicates becomes too low, SAM filters these genes out to control the FDR. In agreement with this hypothesis SAM was perfectly able to identify down-regulation when the full data set was used (data not shown).

The gene selection method proposed in the present work does not provide a direct control on the FDR, but the significance level has been proven to be a direct estimate of the FPR. Thus, if a significance level of 0.001 is used and 12488 probe sets are displayed on the MG-U74Av2 chip, 12–13 genes are expected to be selected by chance in cases where all genes are in fact not differentially expressed. Therefore, a researcher can test how many genes would be selected over a range of different significance levels and chose the one that results in the most acceptable compromise between number of selected genes and estimated FPR.

## Conclusions

The proposed DEG identification method provides a direct control of the FPR and an indirect control of the FDR. Moreover, as tested on the Latin Square data set, our method improved the specificity vs. sensitivity trade-off in comparison to other commonly applied DEG selection techniques. It finally showed an increased robustness when different replicates or numbers thereof are analyzed, giving consistent results even in data sets containing single samples. In conclusion, the global error model presented here may facilitate the analysis of microarray gene expression data by discriminating information from noise, and thus possibly helping the formulation of new hypothesis concerning gene functions.

## Methods

### Data sets

#### 16iDC

RNA was harvested from ten biological samples of unstimulated immature mouse dendritic cells (DC), each extracted from an independent batch of cells. One operator prepared the biotin-labeled cRNA for hybridization from three of the ten RNA samples, a second operator prepared the remaining seven. While operator 1 applied the total RNA protocol to all of its three samples, operator 2 applied the purified mRNA protocol to five of its seven samples and the total RNA protocol to the remaining two. Two of the three cRNA samples prepared by operator 1 and four of the seven cRNA samples prepared by operator 2 have been hybridized twice; therefore, a total of 16 MG-U74Av2 GeneChips (Affymetrix, Santa Clara, CA) have been employed.

#### Leigh syndrome

Eight RNA samples were harvested from human fibroblast cell lines each deriving from a distinct Leigh syndrome patient [19,20] and individually hybridized on HG-U133A GeneChips (Affymetrix).

#### Muscle biopsies

Four individual and two pooled RNA samples from human muscle biopsies of sixteen healthy young male donors were hybridized on six HG-U95Av2 GeneChips (Affymetrix). This data set was downloaded from [21], experiment code: GSE80 [22].

#### Latin Square

This data set consists of 3 technical replicates of 14 separate hybridizations (named Exp01–14) of 42 spiked transcripts in a complex human background at concentrations ranging from 0.125 pM to 512 pM. Thirty of the spikes are isolated from a human cell line, four spikes are bacterial controls, and eight spikes are artificially engineered sequences believed to be unique in the human genome. Further details on the design of the Latin Square data set can be found at [23]. Considering the redundancy of some probe sets, there are a total of 62 distinct probe sets designed to match the 42 spiked transcripts.

#### 16iDC+LPS

This data set consists of the same samples of the 16iDC data set, but includes additional four samples as a second experimental condition. To this end dendritic cells were stimulated to mature with lipopolysaccharide (LPS) for 24 hours. Two independent biological samples were harvested and individually processed by the same two operators that prepared the samples for the 16iDC data set: one applied the total RNA protocol, the other one applied the purified mRNA protocol. Each cRNA sample was hybridized twice, thus using a total of four Affymetrix MG-U74Av2 chips.

### Software

All chips mentioned in the present study were hybridized and scanned following Affymetrix recommendations and MicroArray Suite 5.0 (MAS5) was used as the image acquisition and analysis software. All data sets used passed quality control tests and probe set signals were scaled so that the 4%-trimmed mean of all expression values of each chip was equal to a predefined reference intensity (called TGT) following manufacturer's recommendations:

TGT = 100 for MG-U74Av2 and HG-U133A chips and TGT = 500 for HG-U95Av2 chips.

All procedures for fitting PLGEM, for calculating observed PLGEM-based signal-to-noise ratios (STN), for obtaining expected PLGEM-STN through the resampling-based approach and for comparing observed with expected STN values have been implemented as R functions [24] and will be soon submitted for integration into the Bioconductor project [25].

## Authors' contributions

NP conceived the study and drafted the manuscript. MP wrote the software, participated in the design of the study and in the editing of the manuscript. CV performed the microarray experiments, participated in the design of the study and the editing of the manuscript. MC participated in the microarray experiments, AS participated in the design of the algorithms, FG and PRC coordinated the study. All authors read and approved the final manuscript.

## Supplementary Material

Additional File 1 **Performance of modeling method using different combinations of parameters p and q**. The modeling method described in this study was tested on the 16iDC data set using different combinations of partitions (5, 10, 20, 50, 100, 200 and 500), and quantiles (0.01, 0.02, 0.05, 0.1, 0.2, 0.5, 0.8, 0.9, 0.95, 0.98 and 0.99). For all 77 analyzed combinations of p and q regression lines were fitted to the data as described in the text. Goodness of fit was evaluated from the resulting slope (panel A), intercept (panel B) and adjusted r^2 ^(panel C). Click here for file
